# Hyperoside Suppresses Renal Inflammation by Regulating Macrophage Polarization in Mice With Type 2 Diabetes Mellitus

**DOI:** 10.3389/fimmu.2021.733808

**Published:** 2021-12-03

**Authors:** Jialing Liu, Yanmei Zhang, Hongqin Sheng, Chunling Liang, Huazhen Liu, Jose Alberto Moran Guerrero, Zhaoyu Lu, Wei Mao, Zhenhua Dai, Xusheng Liu, Lei Zhang

**Affiliations:** ^1^ State Key Laboratory of Dampness Syndrome of Chinese Medicine, The Second Clinical College of Guangzhou University of Chinese Medicine, Guangzhou, China; ^2^ Nephrology Department, The Second Affiliated Hospital of Guangzhou University of Chinese Medicine, Guangzhou, China; ^3^ Department of Biochemistry, School of Medicine, Southern University of Science and Technology, Shenzhen, China; ^4^ Section of Immunology and Joint Immunology Program, Guangdong Provincial Academy of Chinese Medical Sciences, Guangzhou, China; ^5^ Monterrey Institute of Technology, Tecnológico, Monterrey, Mexico; ^6^ Guangdong Provincial Key Laboratory of Clinical Research on Traditional Chinese Medicine Syndrome, Guangzhou, China; ^7^ Guangdong-Hong Kong-Macau Joint Lab on Chinese Medicine and Immune Diseases, Guangzhou University of Chinese Medicine, Guangzhou, China

**Keywords:** diabetic nephropathy, macrophage polarization, hyperoside, renal inflammation, Th1 cell, Treg cell

## Abstract

Accumulating evidence reveals that both inflammation and lymphocyte dysfunction play a vital role in the development of diabetic nephropathy (DN). Hyperoside (HPS) or quercetin-3-O-galactoside is an active flavonoid glycoside mainly found in the Chinese herbal medicine Tu-Si-Zi. Although HPS has a variety of pharmacological effects, including anti-oxidative and anti-apoptotic activities as well as podocyte-protective effects, its underlying anti-inflammatory mechanisms remain unclear. Herein, we investigated the therapeutic effects of HPS on murine DN and the potential mechanisms responsible for its efficacy. We used C57BLKS/6J ^Lep^ *db/db* mice and a high glucose (HG)-induced bone marrow-derived macrophage (BMDM) polarization system to investigate the potentially protective effects of HPS on DN. Our results showed that HPS markedly reduced diabetes-induced albuminuria and glomerular mesangial matrix expansion, accompanied with a significant improvement of fasting blood glucose level, hyperlipidaemia and body weight. Mechanistically, pretreatment with HPS effectively regulated macrophage polarization by shifting proinflammatory M1 macrophages (F4/80^+^CD11b^+^CD86^+^) to anti-inflammatory M2 ones (F4/80^+^CD11b^+^CD206^+^) *in vivo* and in bone marrow-derived macrophages (BMDMs) *in vitro*, resulting in the inhibition of renal proinflammatory macrophage infiltration and the reduction in expression of monocyte chemoattractant protein-1 (MCP-1), tumor necrosis factor (TNF-α) and inducible nitric oxide synthase (iNOS) while increasing expression of anti-inflammatory cytokine Arg-1 and CD163/CD206 surface molecules. Unexpectedly, pretreatment with HPS suppressed CD4^+^ T cell proliferation in a coculture model of IL-4-induced M2 macrophages and splenic CD4^+^ T cells while promoting their differentiation into CD4^+^IL-4^+^ Th2 and CD4^+^Foxp3^+^ Treg cells. Taken together, we demonstrate that HPS ameliorates murine DN *via* promoting macrophage polarization from an M1 to M2 phenotype and CD4^+^ T cell differentiation into Th2 and Treg populations. Our findings may be implicated for the treatment of DN in clinic.

## Introduction

Diabetic nephropathy (DN) is a serious microvascular complication in patients with diabetes mellitus (DM) and is increasingly regarded as an inflammatory process (1–3). Macrophages are important cells involved in the initiation of inflammatory responses and play a critical role in the pathogenesis of DN by secreting various proinflammatory mediators. They can differentiate into proinflammatory M1 macrophages through the classical activation and anti-inflammatory M2 macrophages *via* the alternative activation (4). In addition, Th1, Th2, Th17 and cytotoxic T cells are also involved in the development and progression of DN (5, 6). Maintaining the M1/M2, Th1/Th2 and Th17/Treg immune balances can reduce the production of proinflammatory cytokines and improve DN (7). Therefore, we hypothesized that the imbalance in both innate (M1/M2) and adaptive immunities (Th1/Th2 and Th17/Treg) could play a crucial role in the pathogenesis of DN, while rebalancing these immune responses might represent a novel approach for the treatment of DN.

As a traditional herbal ingredient, Hyperoside (HPS) is one of the main active components in the Chinese herb Tu-Si-Zi (8) (**Supplementary Figure 1**), which exhibits the anti-inflammatory, anti-oxidant and anti-cancerous properties. Because of its high efficacy and low toxicity, HPS is commonly used in treating a variety of ischemic brain and heart diseases. In recent years, emerging evidence has shown the anti-inflammatory effects of HPS on various diseases, such as non-alcoholic fatty liver disease (9), osteoarthritis (10), DM-induced cognitive dysfunction (11) and pulmonary fibrosis (12) *in vivo*, and on lipopolysaccharide (LPS)-stimulated cell injury in a model of *in vitro* experiments (13–15) by suppressing activation of the NF-κB signaling pathway. Studies have also confirmed that HPS can inhibit the high glucose (HG) induced inflammatory injury *in vitro* and *in vivo*. For example, it can suppress vascular inflammation caused by HG in the human umbilical vein endothelial cells (HUVECs) *in vitro* and in mice (16, 17). And it alleviates HG-induced apoptosis and inflammatory responses of HK-2 cells through the miR-499a-5p/NRIP1 axis (18). However, very few studies have explored the mechanisms underlying the effects of HPS on renal inflammatory injury in diabetic condition. Thus, the effects HPS on DN and its molecular mechanisms of action remain unclear and warrant further investigation.

In the present study, we aimed to investigate the potentially therapeutic effects of HPS on DN and to delineate the mechanisms underlying the therapeutic effects of HPS in a type-2 DN model of *db/db* mice and *in vitro* by focusing on its immunologically regulatory mechanisms responsible for the differentiation and activation of macrophages and CD4^+^ T cells. We found that HPS indeed attenuated DN in a mouse model of type 2 diabetes mellitus by regulating macrophage polarization.

## Materials and Methods

### Antibodies and Reagents

The micro-albumin assay kit was obtained from Abcam Biotechnology (Abcam, Cambridge, UK). Hyperoside with a purity higher than 98% was purchased from Sigma-Aldrich (St Louis, USA) and was suspended in 0.5% carboxymethyl cellulose sodium salt high viscosity (CMCNa) (MP Biomedicals, LLC, USA) solution for animal experiments or dissolved in 0.1% DMSO for cell culture experiments. Glucose was purchased from Sigma-Aldrich (St Louis, MO, USA) while Trizol reagents were manufactured by Invitrogen (California, USA). cDNA Kit was purchased from Promega (Promega Corporation, Madison, WI). 24-well transwell chamber was bought from Corning (Corning, Shenzhen, China). All flow cytometric antibodies, including F4/80, CD11b, CD86, CD206, CD4, IL4, and Foxp3 were purchased from eBioscience or Biolegend. And details of the antibodies used in this study are listed in the **Table 1**.

**Table 1:** Antibodies used for flow cytometry, IHC and IF.

Antibody name	Source	Catalogue number	Dilution
Antibodies used for flow cytometry			
F4/80	Biolegend	123116	(0.5µg)/test
CD11b	BD Pharmingen	560455	(0.5µg)/test
CD86	Biolegend	105005	(0.5µg)/test
CD206 (MMR)	eBioscience	25-2061-82	(0.5µg)/test
IL-4	BD Pharmingen	554389	(0.5µg)/test
CD4	Biolegend	100516	(0.5µg)/test
Foxp3	Biolegend	126419	(0.5µg)/test
Antibodies used for IHC			
Anti-CD68	Abcam	ab201340	1:400
Anti-MCP1	Abcam	ab7202	1:100
HRP conjugated Goat Anti-Mouse IgG (H+L)	Servicebio	GB23301	1:200
HRP conjugated Goat Anti-Rabbit IgG (H+L)	Servicebio	GB23303	1:200
Antibodies used for IF			
IL-17 antibody	Santa Cruz	374218	1:100
Foxp3 antibody	Santa Cruz	53876	1:100
IFN-γ antibody	R&D	AF-585-NA	1:100
Anti-Mannose Receptor antibody	Abcam	ab64693	1:100
Anti-F4/80 antibody	Abcam	ab16911	1:100
Goat Anti-Rabbit IgG H&L (Alexa Fluor^®^ 488) preadsorbed	Abcam	ab150081	1:1000
Cy3-labeled anti-mouse antibody	Beyotime	A0521	1:200
NL557-labeled anti-Goat antibody	R&D	NL001	1:200

### A Mouse Model of Diabetic Nephropathy in *db/db* Mice

Male *db/db* mice (C57BL/KsJ) (19) at the age of 8 weeks were used in this study. The *db/db* mice and heterozygote age-matched *db/m* mice were originally obtained from Model Animal Research Center of Nanjing University, Jiangsu, China and were maintained following guideline “Principles of Laboratory Animal Care and Use in Research” (Ministry of Health, Beijing, China). Animals were placed in a controlled environment of humidity (about 60%) and room temperature (about 24 ± 1°C) with an alternating 12h light and dark cycle. The animals were allowed free access to standard laboratory tap water and food. The mice were then randomly divided into four groups and treated without or with HPS intraperitoneally for 12 consecutive weeks, as described below: 1) Control *db/m* mice (n=6) received the same volume of distilling water; 2) Control *db/db* mice (n=6) were given with the same volume of distilling water; 3) *db/db* +HHPS mice (n=7) received Hyperoside at a high dose of 80mg/kg/day; 4) *db/db* +LHPS mice (n=7) were treated with Hyperoside at a low dose of 40mg/kg/day.

Fasting blood glucose levels were measured twice weekly using the Bayer glucose monitor (Bayer, Bergkamen, Germany). Mice were sacrificed 12 weeks after HPS treatment to determine serum concentrations of total cholesterol (TC) and low-density lipoprotein cholesterol (LDL-C). Serum and urinary creatinine levels were measured using the enzymatic colorimetric method *via* an automatic biochemistry analyzer (Toshiba-120FR, Tokyo, Japan). Urinary albumin concentration was measured using the ELISA kit with an anti-mouse albumin antibody (Cusabio, Wuhan, China) and was normalized to the urinary creatinine levels and expressed as urinary mAlb/Cr. UACR was calculated according to the following equation: UACR (mg/g) = urinary albumin(mg)/urinary creatine(g). All animal experiments were approved by the Institutional Animal Care and Use Committee of Guangzhou University of Chinese Medicine.

### Renal Pathology

The kidney tissues were fixed in 10% formalin buffer and then embedded in paraffin for light microscopic examination. Serial tissue sections (5μm) were stained with hematoxylin & eosin (HE) and periodic acid-Schiff (PAS). Mesangial matrix expansion was determined by assessing PAS-positive materials in the mesangial region. A percentage of the PAS-positive area was analyzed using Image-Pro Plus (Version 5.1.0.20, Media Cybernetics, Silver Spring, MD, USA) and Leica Q500MC image analysis software. Semi-quantitative analysis was performed with 20 glomeruli randomly selected for each subject (at least five mice in each group) and evaluations were made in coded slides.

### Immunohistochemistry

Immunohistochemical staining was performed on paraffin sections using a microwave-based antigen retrieval technique, which involved heat-induced antigen retrieval (HIAR) with sections incubated in ethylenediaminetetraacetic acid (Tris-EDTA) buffer (pH 9.0). The sections were further incubated with the following primary antibodies at 4°C overnight: anti-CD68 (catalog no. ab201340, Abcam) and anti-MCP-1 (catalog no. ab7202, Abcam), followed by the appropriate secondary antibody. The sections were then developed with 3, 3-diaminobenzidine (DAB) to produce a brown product and were counterstained with hematoxylin.

### Immunofluorescence

Frozen renal tissue sections embedded in OCT were fixed in precooled acetone for 15min, blocked with 1%BSA for 30 min and then incubated with IFN-γ (catalog no. AF-585-NA, R&D), IL-17 (catalog no. 374218, Santa Cruz), FoxP3 (catalog no. 53876, Santa Cruz), F4/80 (catalog no. ab16911, abcam), or CD206 (catalog no. ab64693, abcam) at a dilution of 1:100 overnight at 4°C, followed by secondary anti-goat IgG (catalog no. NL001, R&D), Cy3-labeled goat anti-mouse IgG (catalog no. A0521, Beyotime) or goat anti-rabbit IgG Alexa Fluor-488(catalog no. ab150081, abcam). After staining, the cell nuclei were stained with 4’,6-diamidino-2-phenylindole (DAPI), and images were obtained using confocal microscopy(400x).

### Isolation of Bone Marrow-Derived Macrophages (BMDMs) and Induction of M2 Macrophages

Bone marrow-derived macrophages were isolated from male C57/BL6 mice, as previously described (20), using F4/80 (macrophage marker) and CD11b (myeloid marker), and double-positive macrophages were confirmed with a high purity (about 94.9% of F4/80-positive and CD11b-positive cells on day 7 of culture *in vitro*) (**Supplementary Figure 2**). Briefly, 6-week-old mice were sacrificed, and femur and tibia bone marrow cells were flushed into phosphate-buffered saline (PBS) that contained 2% heat-inactivated fetal bovine serum (FBS), followed by centrifuging at 500g for 5min and lysis with RBC lysis buffer (BOSTER, Wuhan, China). The cells were then suspended in 15% L929-conditioned medium (RPMI-1640 medium containing 10% FBS, 1% penicillin/streptomycin, and 15% L929 cell culture supernatant) and left to adhere for 24 hrs at 37°C in an atmosphere of 5% CO2. The adherent cells were re-plated in a six-well plate with 15% L929-conditioned medium at a density of 1x10^6 per well for seven days to obtain BMDMs with the medium being changed every two days. The BMDMs were then exposed to high glucose in the presence or absence of HPS for 48 hrs: (1) Control group (8mM glucose); (2) HG group (35mM glucose); (3) HG+HHPS group (35mM glucose +50uM HPS); (4) HG+MHPS group (35mM glucose +25uM HPS); (5) HG +LHPS group (35mM glucose +12.5uM HPS).

Besides, M2 macrophages were obtained through addition of 20ng/ml of recombinant murine IL-4 (cat no.21414, Peprotech) to the bone marrow cells before incubation at 37°C with 5% CO2 for 36 hrs to induce the differentiation of BMDMs into M2 macrophages.

### Real-Time Reverse Transcriptase-Polymerase Chain Reaction (RT-PCR)

Total RNA was isolated from kidney tissues or HG-induced macrophages *in vitro* using Trizol reagent (Qiagen, San Diego, CA, USA) following the manufacturer’s instructions. 1ug RNA was reversely transcribed to cDNA using Oligo(dT)_15_ primer and superscript reverse transcriptase (Promega Corporation, Madison, WI). Target gene expression was quantified by real-time PCR using SYBR Green Supermix and the ABI Real-Time PCR Reaction System (Bio-Rad Laboratories). PCR conditions were described as the following: denaturation at 95°C for 3 min, followed by 40 cycles at 95°C for 15 s, 57°C for 30 s, and 72°C for 30 s, with final elongation at 72°C for 10 min. A mouse housekeeping gene β-actin was selected as the internal control. Three independent experiments were performed by calculating the relative mRNA levels using ^2-△△Ct^ methods with values normalized to the reference gene β-actin. The primer sequences (Sangon Biotech, Shanghai, China) that were used are listed in **Table 2**.

**Table 2:** The primer sequences used in quantitative real-time PCR.

Gene Primer	Forward Sequence (5’-3’)	Reverse Sequence (5’-3’)
β-actin	GCTTCTTTGCAGCTCCTTCG	GGCCTCGTCACCCACATAG
iNOS	GACATTACGACCCCTCCCAC	GCACATGCAAGGAAGGGAAC
TNF-α	CACCTGGCCTCTCTACCTTG	CGATTACAGTCACGGCTCCC
MCP-1	TTGACCCGTAAATCTGAAGCTAAT	TCACAGTCCGAGTCACACTAGTTCAC
Arg-1	ACATTGGCTTGCGAGACGTA	ATCACCTTGCCAATCCCCAG
IFN-γ	CACGGCACAGTCATTGAAAG	CATCCTTTTGCCAGTTCCTC
IL-10	CCAAGCCTTATCGGAAATGA	TCCTGAGGGTCTTCAGCTTC
IL-17	GTCCAAACACTGAGGCCAAG	ACGTGGAACGGTTGAGGTAG

iNOS, inducible nitric oxide synthase; TNF-α, tumor necrosis factor α; MCP-1, monocyte chemoattractant protein-1; Arg-1, arginase-1; IFN-γ, interferon gamma; IL-10, interleukin 10; IL-17, interleukin 17.

### Purification of Splenic T Cells

The purification of CD3^+^ T cells was carried out by nylon wool columns as described previously (21). Briefly, spleens from 6-week male C57/BL6 mice were gently and mechanically disassociated through a 70um cell strainer and lysed with RBC lysis buffer (BOSTER, Wuhan, China) to obtain splenic cells suspension. Autoclaved and sterile nylon fibers (0.5g) were put into a 10mL column, and then the column was equilibrated by 20mL warm RPMI-1640 medium, sealed and incubated at 37°C and 5% CO2 for 1 hour. Spleen cells (1x10^8) were added to the column, sealed and incubated at 37°C and 5% CO2 for 1 hour. Cells subjected to nylon wool purification were resuspended with 2mL warm RPMI-1640 medium, and the column was washed with 2mL warm RPMI-1640 twice. Finally, cells were collected, centrifuged and resuspended for further experiment.

### Co-Culture of M2 Macrophages and T Cells

Transwell coculture of mouse M2 macrophages and T cells was performed using a 24-well multi-well chamber and polycarbonate membranes (0.4-um porous). Briefly, T cells were collected and seeded in the upper 24-well transwell plate (5x10^4 cells/well) with 10ng/mL IL-2 (Peprotech, USA) and 1x PMA/ionomycin (Multi-Science Biotechnology, China) in complete RPMI-1640 medium, while M2 macrophages were cultured in the lower 24-well transwell plate (5x10^5 cells/well) with complete RPMI-1640 medium in the absence or presence of different concentrations of HPS for 72 hrs. After co-culture, T cells were collected and centrifuged (400g, 4°C, 5min) for flow cytometric analysis.

### Cell Proliferation Assays

T cell proliferation in the coculture was determined using Carboxyfluorescein Diacetate Succinimidyl Ester (CFSE) Cell Proliferation Assay and Tracking Kit (Beyotime, China). For CFSE staining, T cells were isolated, purified and resuspended to 1x10^6 cells/ml in 1x CFSE cell labeling solution at 37°C for 10 min, followed by centrifuging and washing with RPMI-1640 complete medium. After the coculture, CFSE-labeled T cells were collected to perform co-further culture with M2 macrophages for 72 hrs, and finally analyzed using a flow cytometer (Novocyte Quanteon). Data were interpreted as the percentage of proliferated cells.

### Flow Cytometric Analysis

Single-cell suspensions from spleens were prepared as the following. Briefly, spleens from the *db/m* and *db/db* mice were minced and filtered through 40μm nylon meshes, and then the splenic cells were suspended in PBS and lysed with red blood cell-lysis buffer, followed by centrifuging (400g, 4°C) for 5min. As for the bone marrow-derived macrophages (BMDMs), they were first isolated and cultured as described above. Then the splenic T cells and BMDMs were incubated with fluorochrome-conjugated Abs against various surface makers at room temperature for 30 min, including APC-conjugated anti-mouse F4/80 (catalog no. 123116, Biolegend), V450- Rat anti-mouse CD11b (catalog no. 560455, BD), FITC-conjugated anti-mouse CD86 (catalog no. 105005, Biolegend) and PE-Cyanine7-conjugated anti-mouse CD206 (MMR) (catalog no. 25-2061-82, eBioscience). To observe the effects of HPS on the generation of Th2 and Treg cells in a co-culture model of M2 and T cells, T cells were cultured in medium containing 1x BFA/Moensin (Multi-Science Biotechnology, China) for 8 hrs before cells were collected. The T cells were centrifuged, washed and incubated with APC anti-mouse CD4 Ab (catalog no. 100516, Biolegend). For intracellular staining, the cells were permeabilized using FoxP3/Transcription Factor Fixation/Permeabilization Concentrate and Diluent Kit (eBioscience, USA) and then stained with brilliant Violet 421-anti-mouse FoxP3(catalog no. 126419, Biolegend) and PE anti-mouse IL-4 Abs (catalog no.554389, BD). For cell proliferation assays, T cells were first labeled with CFSE before coculture. After 48 hrs of the coculture, T cells were collected, centrifuged, resuspended, and incubated with APC anti-mouse CD4 Ab (catalog no. 100516, Biolegend). Cells finally were analyzed *via* a flow cytometer (Novocyte Quanteon).

### Statistical Analysis

All data were expressed as mean ± standard deviation (SD) and analyzed using Graphpad Prism 8.0 software (San Diego, CA, USA). One-way ANOVA with a one-tailed Student’s t-test was used to identify significant differences in multiple comparisons. The *post hoc* comparisons using the Student-Newman-Keuls tests were performed for inter-group comparisons of multiple variables. A probability of *P* < 0.05 was considered to be statistically significant.

## Results

### Hyperoside (HPS) Reduces Albuminuria and Improves Glycolipid Metabolism Dysfunction in *db/db* Mice

**Figure 1:**
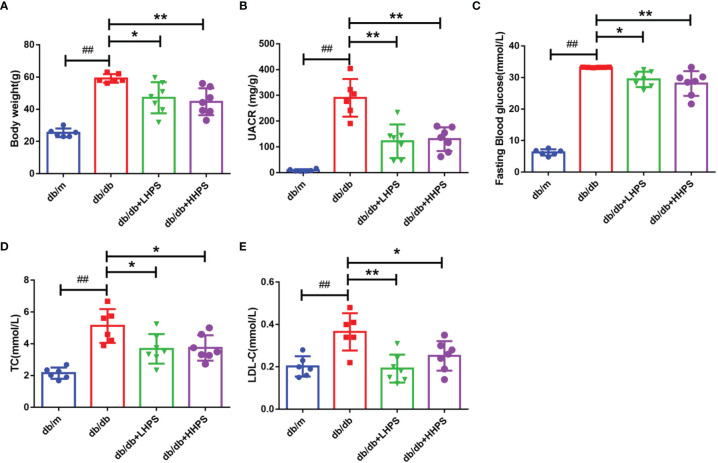
Clinical and metabolic parameters in four groups of mice: *db/m*, *db/db*, *db/db* +LHPS, and *db/db* +HHPS mice. **(A)** Body weight (g), **(B)** Urine albumin-to-creatinine ratio (UACR) (mg/g), **(C)** Fasting blood glucose (FBG) (mmol/L), **(D)** Total cholesterol (TC) (mmol/L), and **(E)** Low density lipoprotein-cholesterol (LDL-C) (mmol/L) were measured after HPS treatment. *db/m*, non-diabetic normal control mice; *db/db*, DN mice treated with distilled water; *db/db*+ LHPS or HHPS, *db/db* mice treated with 40 (LHPS) or 80 mg/kg/day (HHPS) of HPS, respectively. Data are presented as the mean ± SD (n=6-7, with individual donors shown as dots, ^##^ *P* < 0.01 *vs db/m*; *P < 0.05 and **P < 0.01 *vs db/db*).

We first determined the effects of HPS on proteinuria as well as glycolipid metabolism in *db/db* mice. Compared with control *db/m* mice, the levels of urine albumin-creatine ratio (UACR), body weight (BW), fasting blood glucose (FBG), total cholesterol (TC), and low-density lipoprotein-cholesterol (LDL-C) were significantly higher in *db/db* mice (**Figure 1**). Administration of HPS (either 40 or 80mg/kg/day) for 12 weeks resulted in a significant reduction of these indicators in *db/db* mice (**Figure 1** and **Table 3**).

**Table 3:** The effects of Hyperoside pre-treatment on clinical and metabolic parameters of *db/db* mice.

Index	*db/m*	*db/db*	*db/db* +HHPS	*db/db* +LHPS
BW(g)	25.38 ± 2.68	59.18 ± 2.60^##^	44.66 ± 8.39^**^	47.14 ± 9.67^*^
UACR (mg/g)	8.93 ± 4.14	290.33 ± 73.20^##^	129.61 ± 45.85^**^	121.89 ± 65.09^**^
FBG (mmol/L)	6.27 ± 0.98	33.22 ± 0.08^##^	28.53 ± 4.31^**^	29.41 ± 2.42^*^
TC (mmol/L)	2.15 ± 0.36	5.12 ± 4.22^##^	3.49 ± 3.32^*^	3.74 ± 3.51^*^
TG (mmol/L)	1.37 ± 0.95	1.62 ± 0.81	1.97 ± 0.85	1.95 ± 0.74
LDL-C (mmol/L)	0.20 ± 0.05	0.37 ± 0.09^##^	0.25 ± 0.07^*^	0.19 ± 0.07^**^
HDL-C(mmol/L)	1.08 ± .0.21	1.99 ± 0.42	1.90 ± 0.39	1.97 ± 0.47
BUN (mmol/L)	9.89 ± 2.21	9.42 ± 1.96	8.51 ± 1.86	8.61 ± 1.85
SCr (μmol/L)	10.00 ± 1.90	8.40 ± 3.36	9.38 ± 2.07	8.50 ± 3.30
UA (μmol/L)	224.30 ± 64.48	317.40 ± 97.57^#^	352.25 ± 64.67	376.50 ± 75.92
ALB (g/L)	42.12 ± 3.14	46.04 ± 8.51	44.23 ± 8.49	45.18 ± 2.38

db/m: normal mice; db/db: treated with vehicle solution; LHPS and HHPS: db/db mice were treated with 40 or 80 mg/kg/d Hyperoside for 12 weeks after DM establishment respectively. Data are presented as mean ± SD. n=6-7. ^#^P < 0.05 vs db/m mice; ^##^P < 0.01 vs db/m mice; *P < 0.05 vs db/db mice; **P < 0.01 vs db/db mice.

BW, body weight; UACR, urine albumin creatine ratio; FBG, fasting blood glucose; TC, total cholesterol; TG, Triglyceride; LDL-C, low density lipoprotein cholesterol; HDL-C, high density lipoprotein cholesterol; BUN, blood urea nitrogen; SCr, serum creatinine; UA, uric acid; ALB, serum albumin.

### HPS Improves Renal Morphological Abnormalities in *db/db* Mice

**Figure 2:**
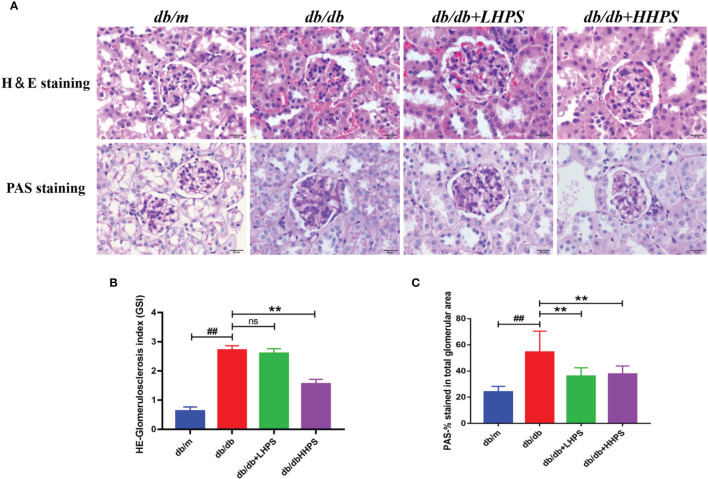
Hyperoside ameliorates mesangial matrix expansion in glomeruli of *db/db* mice. The *db/db* mice were treated with HPS, while control mice receiving vehicle treatment were administered with the same volume of vehicle used to prepare HPS. **(A)** Renal morphology was analyzed by H&E staining on kidney sections while glomerular mesangial matrix fractions and glomerulosclerosis were analyzed using PAS staining in mice at the age of 12 weeks. Figures for representative sections are shown at ×400 magnification. **(B)** Shown is quantification of the glomerulosclerosis index (GSI) for each group. **(C)** The PAS stained areas for each group are also quantified. Values (mean ± SD) were obtained from 3 animals in each group (n=3 per group). Differences between experimental groups were evaluated using ANOVA (^##^ *P* < 0.01 *vs db/m*; **P < 0.01 *vs db/db;* ns, not significant). *db/m*, non-diabetic normal mice; *db/db*, diabetic nephropathy mice; *db/db* +LHPS, diabetic nephropathy group mice treated with a low dose of HPS; *db/db*+HHPS, diabetic nephropathy group mice treated with a high dose of HPS; H&E, Hematoxylin and Eosin; PAS, Periodic Acid–Schiff.

Mesangial matrix expansion and glomerular basement membrane thickening were observed in the kidneys of *db/db* mice and were ameliorated in HPS-treated *db/db* mice (**Figure 2A**). The mesangial expansion index and glomerulosclerosis index, indicating the progression of the mesangial changes in DN, were significantly increased in *db/db* mice compared to *db/m* control mice. However, HPS treatment for 12 weeks significantly decreased both the mesangial expansion index and glomerulosclerosis index, resulting in a reduction of renal mesangial expansion and extracellular matrix accumulation in *db/db* mice (**Figures 2B, C**).

### HPS Treatment Reduces Total Macrophages and Chemokine MCP-1 in *db/db* Mice

**Figure 3:**
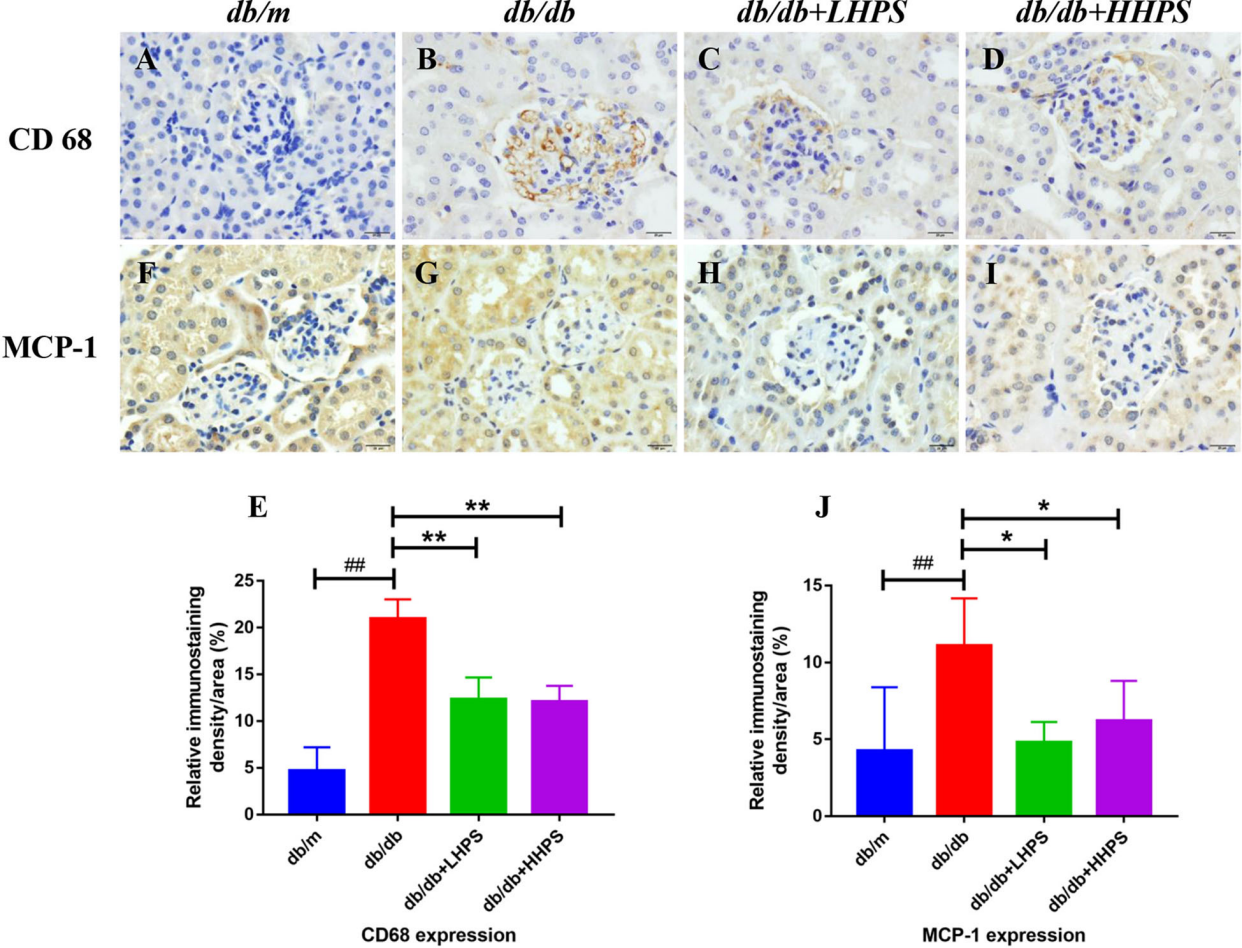
Immunohistochemical staining of CD68 and MCP-1 in glomeruli and interstitial areas (×400, bar=20um) of the kidney in *db/db* mice. Immunohistochemical analysis of CD68-positive **(A–D)** and MCP-1-positive cells **(F–I)** was performed after HPS treatment. The color was developed by incubating with diaminobenzidine and counterstaining with hematoxylin (Magnification x 400). Shown also is quantification of the CD68+ **(E)** or MCP-1+ area **(J)**. The data are presented as the mean ± SD (n = 5 per group, ^##^ *P* < 0.01 *vs db/m*; *P < 0.05 and **P < 0.01 *vs db/db*, with each symbol color representing a particular group).

To determine an impact of HPS on total macrophage numbers in the kidney of *db/db* mice, immunohistochemistry (IHC) staining was performed. We found that compared to control *db/db* mice, administration of HPS remarkedly decreased the number of CD68-positive macrophages in the renal tissue, although they were significantly increased in control *db/db* mice compared to non-diabetic *db/m* control mice (**Figures 3A–E**). On the other hand, IHC staining also showed that HPS significantly reduced MCP-1 expression in the kidney tissue of *db/db* mice (**Figures 3F–J**), indicating that overall, HPS is anti-inflammatory in murine DN.

### HPS Alters the Balance of Pro-Inflammatory and Anti-Inflammatory Cytokines in *db/db* Mice

**Figure 4:**
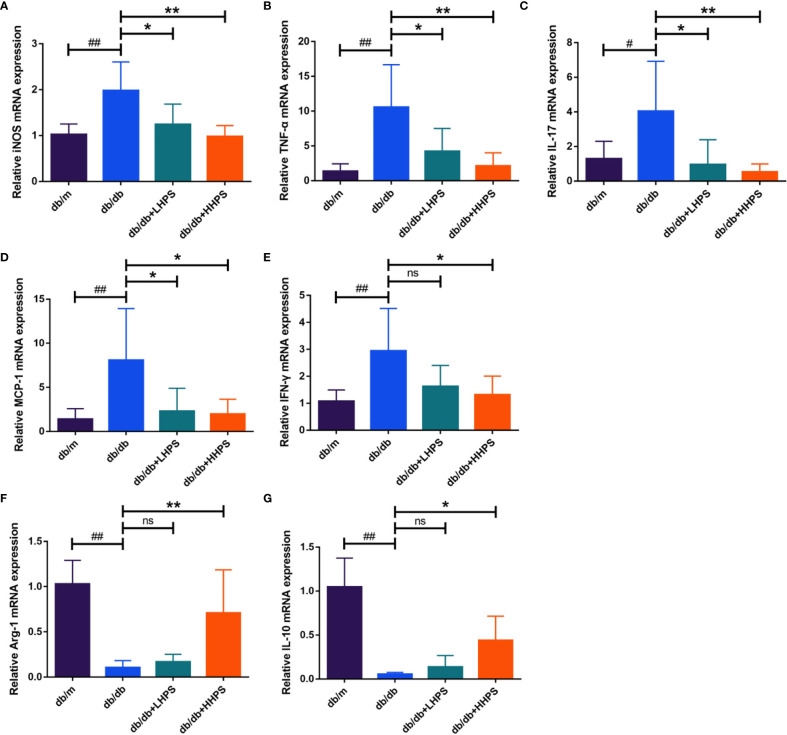
Hyperoside reduces the expression of proinflammatory cytokines while increasing the expression of anti-inflammatory cytokines in the kidney. Quantitative real-time PCR analysis of the proinflammatory cytokines iNOS **(A)**, TNF-α **(B)**, IL-17 **(C)**, MCP-1 **(D)** and IFN-γ **(E)**, and anti-inflammatory cytokines Arg-1 **(F)** and IL-10 **(G)** in the kidney tissue in four groups following HPS treatment: *db/m*, *db/db*, *db/db* +LHPS, and *db/db* +HHPS mice. Relative mRNA expression levels were normalized to β-actin. Data are representatives of three independent experiments and presented as means ± SD (n = 6 per group, ^#^ *P* < 0.05 and ^##^ *P* < 0.01 *vs db/m*; *P < 0.05 and **P < 0.01 *vs db/db;* ns, not significant).

Quantitative real-time PCR analysis was performed to measure the gene expression of the inflammatory cytokines and chemokines in the kidney of *db/db* mice. We found that HPS treatment decreased the gene expression of the pro-inflammatory cytokines/chemokines, including iNOS, MCP-1, IFN-γ, IL-17 and TNF-α, in the renal tissue, whereas it increased the expression of anti-inflammatory cytokines Arg-1 and IL-10 (**Figure 4**). Our results suggest that HPS indeed exerts anti-inflammatory effects in a murine model of DN.

### HPS Promotes M2 Macrophage Polarization in the Kidney of *db/db* Mice

**Figure 5:**
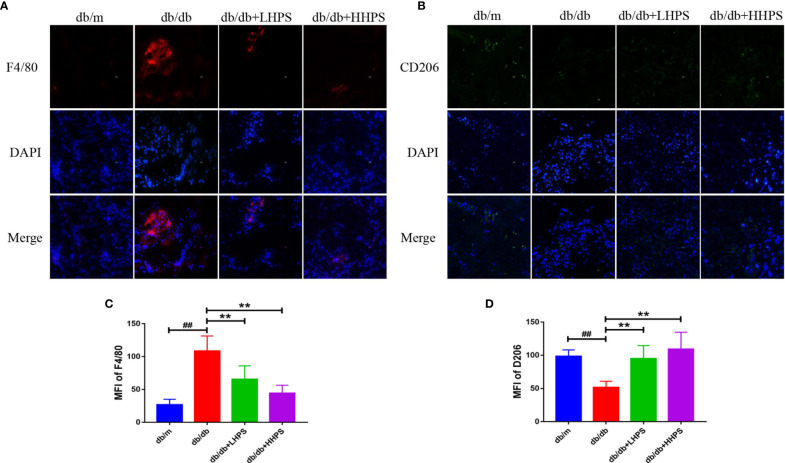
The M2 macrophage polarization in the kidney of *db/db* mice treated with Hyperoside. Immunofluorescence staining showed that Hyperoside promoted the polarization of M2 macrophages and reduced total number of macrophages in the kidney. **(A)** Macrophages were stained for F4/80 (red) in glomeruli of *db/db* mice by immunofluorescence after Hyperoside treatment. **(B)** M2 macrophages were stained for CD206 (green) in glomeruli of *db/db* mice by immunofluorescence. DAPI was used to stain the cellular nucleus. (Original magnification: **×**400. Scale bars = 50 μm). Shown is also quantification of F4/80 **(C)** and CD206 expression **(D)**, as presented as MFI. (n=5 per group, ^##^ *P* < 0.01 vs *db/m*; **P < 0.01 vs *db/db*).

Immunofluorescence staining was performed to determine the effects of HPS on M2 macrophage polarization in the kidney of *db/db* mice since M2 macrophages play an important role in controlling renal inflammation. The results showed that expression of F4/80, a marker of total macrophage population, was increased in the kidney of *db/db* mice compared to *db/m* mice while HPS treatment significantly reduced its expression (**Figures 5A, C**). On the other hand, the downregulated expression of M2 macrophage marker CD206 in the kidney of *db/db* mice was markedly increased by HPS (**Figures 5B, D**). The results indicate that HPS reduces renal inflammatory injury in *db/db* mice *via* promoting M2 macrophage polarization.

### HPS Promotes Splenic M2 Macrophage Generation in *db/db* Mice

**Figure 6:**
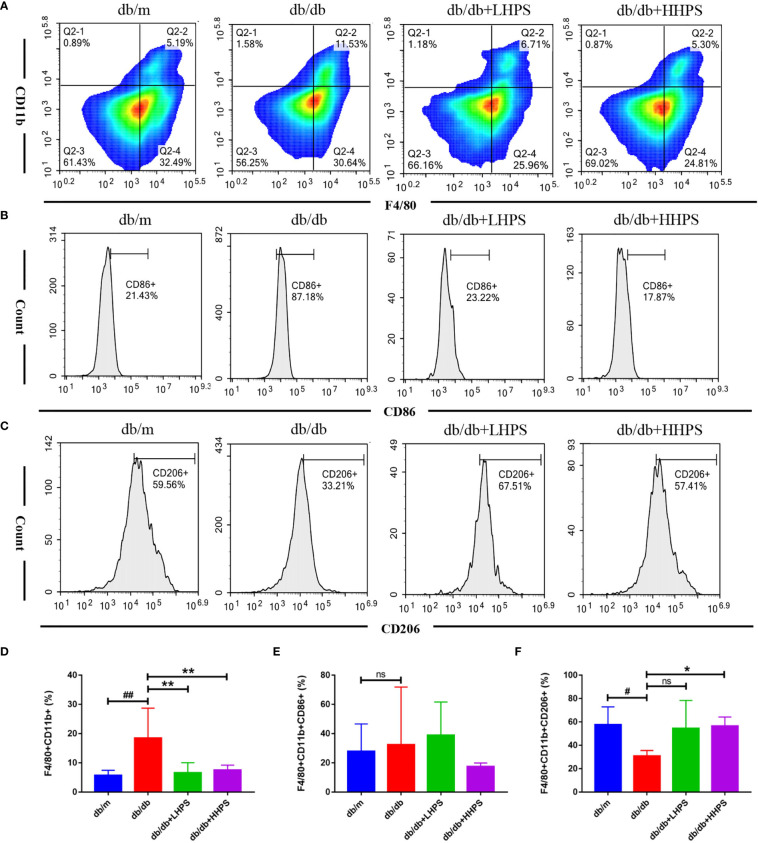
The M2 macrophage polarization in the spleen of *db/db* mice treated with Hyperoside. After HPS treatment, the phenotypes and percentages of MØ (F4/80^+^CD11b^+^) **(A, D)**, M1 (CD86^+^ gated on F4/80^+^CD11b^+^) **(B, E)** and M2 (CD206^+^ gated on F4/80^+^CD11b^+^) macrophages **(C, F)** in the spleen of *db/db* mice were determined *via* flow cytometry. Data are presented as mean ± SD (n = 5 per group, ^#^ *P* < 0.05 and ^##^ *P* < 0.01 *vs db/m*; *P < 0.05 and **P < 0.01 *vs db/db*; ns, not significant).

Flow cytometric analysis showed that HPS reduced the total population of splenic MØ macrophages (F4/80^+^CD11b^+^) in both *db/db* +LHPS and *db/db* +HHPS mice (**Figures 6A, D**). The decreased percentage of F4/80^+^CD11b^+^CD206^+^ cells in spleen of *db/db* mice was significantly increased after HHPS treatment (**Figures 6C, F**). We also found a downward trend for the percentage of F4/80^+^CD11b^+^CD86^+^ cells in *db/db* mice treated with HHPS. However, there were no statistically significant changes in the percentage of F4/80^+^CD11b^+^CD86^+^ cells in control *db/db* mice, compared to *db/m* control mice or HPS treated *db/db* mice (**Figures 6B, E**). These data indicate that HPS promotes splenic M2 macrophage generation in *db/db* mice.

### HPS Also Reduces Protein Expression of Proinflammatory Cytokines Associated With CD4+ T-Cells but Increases FoxP3 Expression in the Kidney of *db/db* Mice

**Figure 7:**
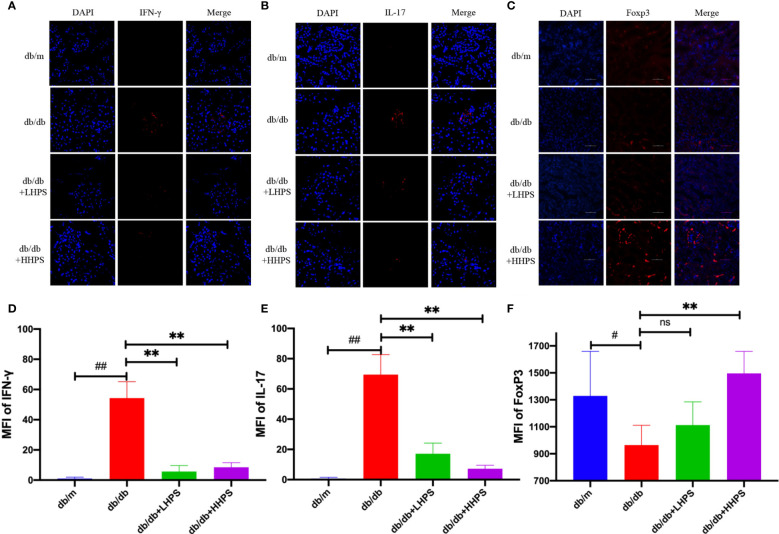
The effects of Hyperoside on the imbalance of T cell differentiation in the kidney. Immunofluorescence staining shows the expressions of CD4+ T-cell associated cytokines, IFN-γ [red, **(A)**], IL-17 [red, **(B)**], and Foxp3 [red, **(C)**] in kidney tissue of *db/db* mice treated with HPS. Quantitative analyses of IFN-γ **(D)**, IL-17 **(E)**, and Foxp3 **(F)** expressions in kidney tissue were also performed (Original magnification: ×400, scale bar = 50 μm). Values are presented as the means ± SD. Differences between experimental groups were evaluated using ANOVA. (n = 5 per group, ^#^ *P* < 0.05 and ^##^ *P* < 0.01 *vs db/m*; **P < 0.01 *vs db/db*; ns, not significant).

To explore whether HPS restores the balance of Th cell subsets or cytokines, we investigated the biological effects of HPS on the protein expression of IFN-γ, IL-17 and FoxP3 in the renal tissue using immunofluorescence (IF) staining. We found that HPS treatment significantly downregulated IFN-γ and IL-17 expressions (**Figures 7A, B**) but upregulated the expression of FoxP3 in the kidney of *db/db* mice compared to that of control *db/db* mice (**Figure 7C**). The same results were confirmed when MFI was analyzed statistically (**Figures 7D–F**), indicating that HPS can alter the balance of Th1/Th17/Treg cells.

### HPS Alters the Balance of Pro-Inflammatory and Anti-Inflammatory Cytokines in BMDMs *In Vitro* Under High Glucose Condition

**Figure 8:**
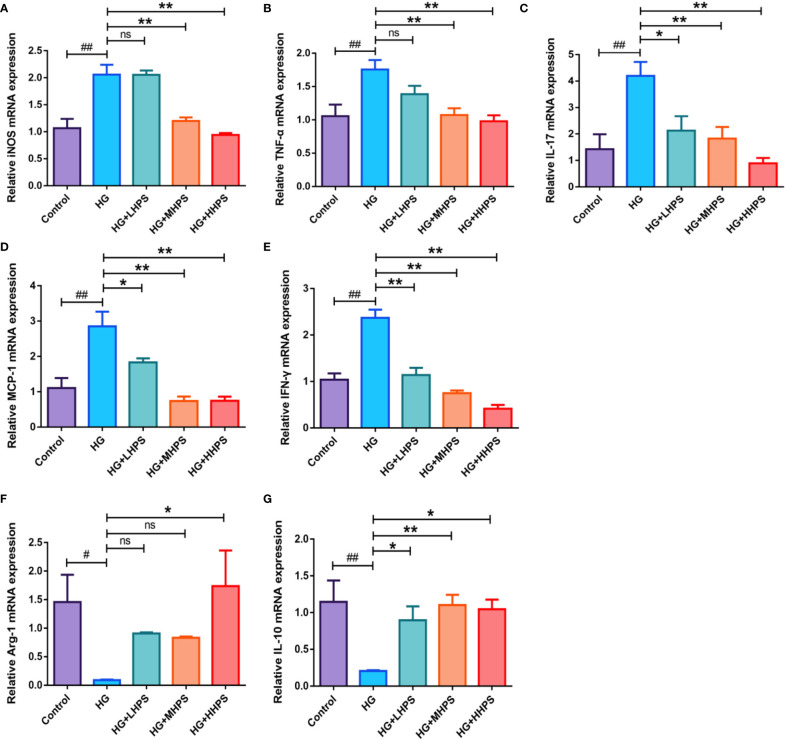
Effects of Hyperoside on the gene expressions of the proinflammatory and anti-inflammatory cytokines in high glucose (HG)-induced BMDMs. BMDMs were treated with high glucose (35mM) for 48 hrs, followed by pretreatment with high-dose HPS (HHPS) (50uM) or low-dose HPS (LHPS) (12.5uM), and then related mRNA expression was determined by qRT-PCR. Relative mRNA expressions of the proinflammatory cytokines iNOS **(A)**, TNF-α **(B)**, IL-17 **(C)**, MCP-1 **(D)** and IFN-γ **(E)**, and anti-inflammatory cytokines Arg-1 **(F)** and IL-10 **(G)** were shown. Values are presented as means ± SD with n = 5 per group. Differences between experimental groups were evaluated using ANOVA (^#^ *P* < 0.05 and ^##^ *P* < 0.01 *vs* Control; *P < 0.05 and **P < 0.01 *vs* HG; ns, not significant). HG, high glucose treated group; HG+LHPS, high glucose and low dose of HPS treatment group; HG+MHPS, high glucose and middle dose of HPS treatment group; HG+HHPS, high glucose and high dose of HPS treatment group.

To examining whether HPS could reverse the inflammatory responses *in vitro*, bone marrow-derived macrophages (BMDMs) were incubated and divided into five groups: (1) Control group (8mM glucose); (2) HG group (35mM glucose); (3) HG+HHPS group (35mM glucose +50uM HPS); (4) HG+MHPS group (35mM glucose +25uM HPS); (5) HG+LHPS group (35mM glucose +12.5uM HPS). Compared with the control group (low glucose), upregulation of pro-inflammatory cytokines, including iNOS, TNF-α, IL-17, MCP-1 and IFN-γ (**Figures 8A–E**), and downregulation of anti-inflammatory cytokines Arg-1 and IL-10 (**Figures 8F, G**) were observed in HG group, indicating that HG can trigger an inflammatory response *in vitro*, mimicking a diabetic condition *in vivo*. Compared with the HG group, however, HG plus HPS groups reduced mRNA levels of M1 macrophage-associated pro-inflammatory mediators iNOS, TNF-α, IL-17, MCP-1 and IFN-γ (**Figures 8A–E**), while the mRNA levels of anti-inflammatory cytokines Arg-1 and IL-10 (**Figures 8F, G**) were increased in the group with high concentrations of HPS. These data suggest that HPS can diminish pro-inflammatory responses stimulated by HG in BMDMs.

### Hyperoside Promotes M2 Macrophage Formation in High Glucose (HG)-Induced BMDMs

**Figure 9:**
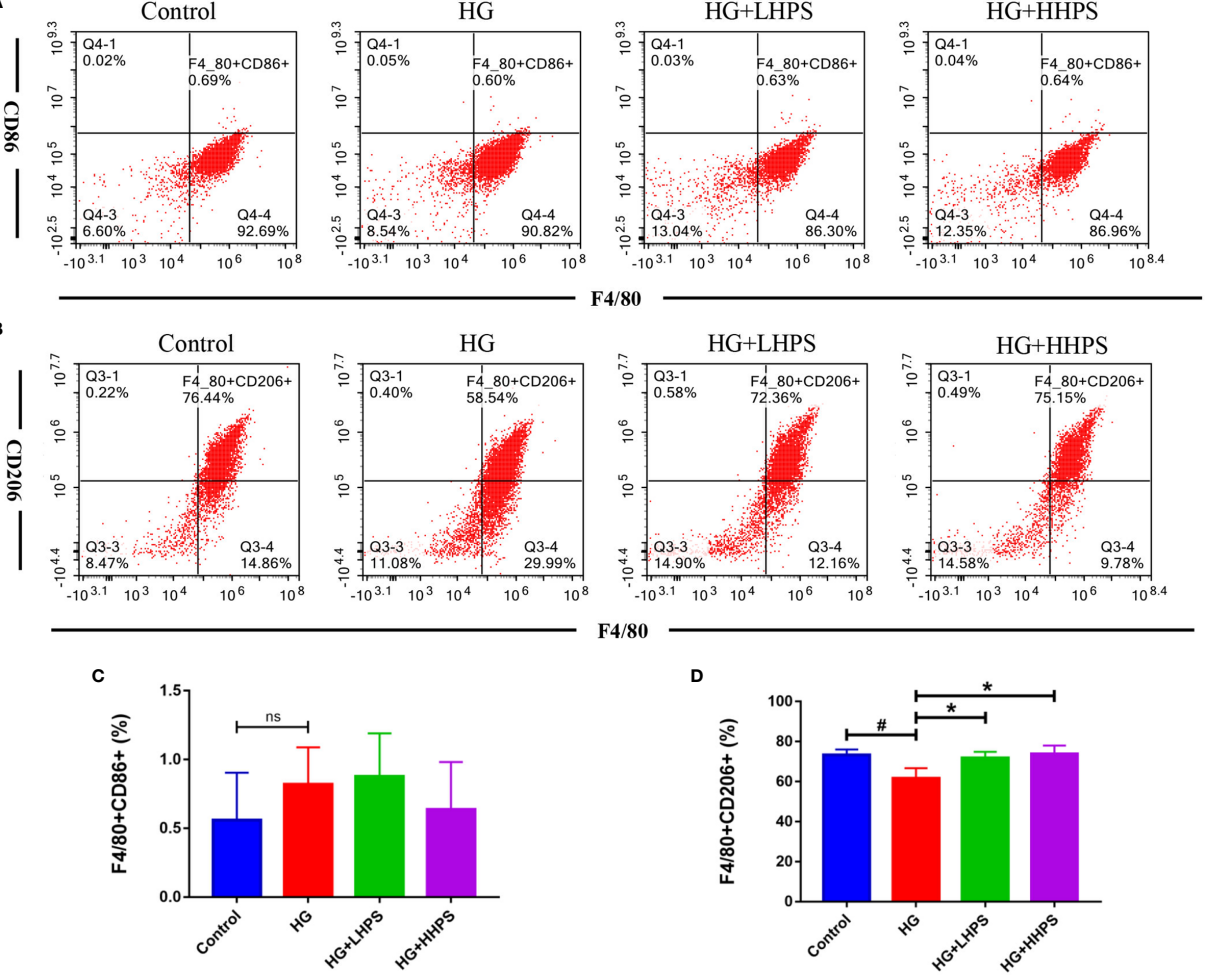
Hyperoside promotes M2 macrophage polarization in high glucose (HG)-induced BMDMs *in vitro.* BMDMs were treated with high glucose (35mM) for 48 hrs, followed by pretreatment with high-dose HPS (HHPS) (50uM) or low-dose HPS (LHPS) (12.5uM), and then collected for flow cytometric analysis. The results showed that there was no significant difference in the proportion of F4/80^+^/CD86^+^ macrophages between groups with or without Hyperoside treatment **(A, C)**. However, the proportion of F4/80^+^/CD206^+^ macrophages was decreased in BMDMs with high glucose but increased after Hyperoside treatment **(B, D)**. Differences between experimental groups were evaluated using ANOVA (^#^ *P* < 0.05 *vs* Control; *P < 0.01 *vs* HG; ns, not significant). HG, high glucose treated group; HG+LHPS, high glucose and low dose of HPS treatment group; HG+HHPS, high glucose and high dose of HPS treatment group.

We then examined the effects of HPS on macrophage polarization *in vitro*. BMDMs were pretreated with HG to mimic the *in vivo* diabetic condition. M1/M2 macrophage polarization was determined *via* flow cytometry and represented as mean fluorescence intensity (MFI). The percentage of F4/80^+^/CD206^+^ BMDMs was significantly decreased in the presence of HG, but increased after HPS treatment (**Figures 9B, D**), indicating that HPS promotes M2 macrophage polarization, which otherwise is inhibited by HG. Meanwhile, we found that the proportion of F4/80^+^/CD86^+^ cells had an upward trend in HG-induced BMDMs and a downward one with a high concentration of HPS treatment (HHPS) (**Figures 9A, C**), yet without a statistical significance. These results indicate that the promotion of M2 macrophage formation may be critical for HPS mediated anti-inflammatory effects in BMDMs in the face of HG.

### HPS Inhibits T Cell Proliferation and Drives T Cell Differentiation Towards Th2 and Treg Populations in a Co-Culture Model of T Cells/M2 Macrophages

**Figure 10:**
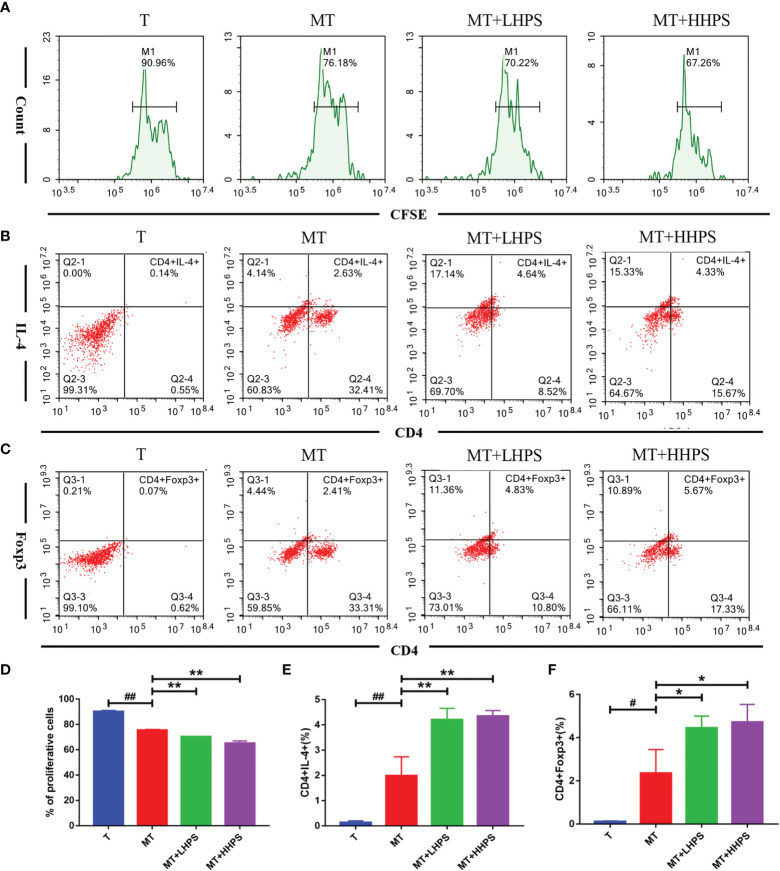
Hyperoside suppresses T cell proliferation while increasing Th2 or Foxp3+ Treg generation in the co-culture with M2 macrophages. T cells cocultured with M2 macrophages at a ratio of 1:10 (T cell/M2 macrophages) were treated with high-dose HPS (HHPS) (50uM) or low-dose HPS (LHPS) (12.5uM) for 72hrs, and then analyzed *via* flow cytometry. **(A, D)** For proliferation assays, T cells were first labeled with CFSE before the co-culture, and the CFSE dilution of CFSE-labeled CD4^+^T cells was detected by flow cytometry. Flow cytometric analysis showed T-cell proliferation was inhibited by addition of M2 macrophages to the coculture system, and the inhibition was further enhanced by HPS. **(B, E)** The percentage of IL4-expressing CD4^+^ T cells was increased by HPS treatment compared with a control group (MT). **(C, F)** The percentage of CD4^+^Foxp3^+^ T cell subsets was also increased by HPS treatment compared with a control group (MT). Differences between experimental groups were evaluated using ANOVA (^#^ *P* < 0.05 and ^##^ *P* < 0.01 *vs* T group; *P < 0.05 and **P < 0.01 *vs* MT group). T, T cells alone; MT, T cells cocultured with M2 macrophages; MT+LHPS, T cells cocultured with M2 macrophages treated with low-dose HPS (12.5 μM); MT+HHPS, T cells cocultured with M2 macrophages treated with high-dose HPS (50 μM).

Given that M2 macrophages affect Treg/Th cell differentiation, we examined the effect of HPS on T cell proliferation and Th2/Treg generation in the coculture of T cells and M2 BMDMs. As shown in the **Figure 10**, T-cell proliferation was inhibited by addition of M2 macrophages to the coculture (MT) as compared with T cell culture alone (T) without M2 macrophages (**Figures 10A, D** **)**, while compared with the coculture group of M2 plus T cells (MT), HPS showed more effective inhibition of T cell proliferation (**Figures 10A, D**). Hence, these results indicate that M2 macrophages suppress T cell proliferation and that HPS plus M2 macrophages can further enhance their suppression of T-cell proliferation. Furthermore, we asked whether HPS also affected the differentiation of T cells co-cultured with M2 macrophages. As shown in **Figure 10**, more T cells co-cultured with M2 macrophages expressed Th2 cytokine IL-4 (CD4^+^IL4^+^) (**Figures 10B, E**) and FoxP3 (CD4^+^Foxp3^+^) (**Figures 10C, F**) than T cells without M2 macrophages.

Interestingly, compared with the M2 and T cell coculture group (MT) alone, the groups treated with HPS (MT+LHPS or +HHPS) showed further upregulation of the frequency of Th2 (CD4^+^IL4^+^) (**Figures 10B, E**) and CD4^+^Foxp3^+^ Treg cells (**Figures 10C, F**). These results indicate that HPS may enhance the ability of M2 macrophages to suppress inflammation by promoting T cell differentiation into Th2/Treg subsets.

## Discussion

Proteinuria or albuminuria is a clinical risk of the onset and development of diabetic nephropathy (DN) (22, 23). The results of this present study showed that Hyperoside (HPS) dramatically attenuated albuminuria, which is usually accompanied by dyslipidemia and obesity in *db/db* mice. We also investigated the mechanisms underlying effects of HPS on DN and revealed that HPS regulated inflammation by promoting M2 macrophage polarization in addition to shifting Th cell balance towards Treg and Th2 cells, thus attenuating the pathogenesis of DN. This study provides a rationale for developing HPS as a drug for the treatment of DN.

In this study, a significantly higher urine albumin-to-creatinine ratio (UACR), hyperglycemia, and an increase in body weight, LDL-C and TC in *db/db* mice suggested that the animals developed DN. When compared with *db/db* mice, significant decreases in UACR were observed in HPS-treated *db/db* mice, suggesting that HPS ameliorated proteinuria in diabetic mice. In addition, the levels of fast blood glucose, LDL-C, TC, and body weights were significantly reduced after treatment with HPS, indicating that it exerted an effect on hyperglycemia and hyperlipidemia. However, the parameters of the renal function, such as Scr and BUN, were not significantly improved by HPS. The effects of HPS on hyperglycemia, hyperlipidemia and renal function index is not totally consistent with the report by Zhang J et al (24), which showed that HPS decreased the Scr in DN mice, although it did not affect the glucose and lipid metabolism. This discrepancy may be due to the differences in animal models used in these two studies. The *db/db* mice used in our study have a background of C57BLKS/J strain, which is a widely used mouse model for type 2 diabetes and an approved model of albuminuria associated with DN, and the renal function in these diabetic mice declines at 15-18 weeks without a significant change of serum biochemical indicators, whereas Zhang et al. used low-dose of STZ to induce type 1 diabetes in rats, resulting in obvious deterioration in serum renal function indicators. In the present study, we confirmed the potential protective effects of HPS on glomerulosclerosis in DN. When *db/db* mice were treated with HPS, their histopathology was remarkably improved, which was still consistent with the findings by Zhang L et al (25) that HPS was involved in the epigenetic regulation of glomerulosclerosis.

Our results demonstrated that HPS exerted an anti-inflammatory effect on DN by regulating macrophage polarization. Macrophages are one of the most abundant innate immune cells in renal tissue of individuals with DN. Activation of macrophages plays a key role in renal inflammatory injury in DN (26). Recently, the role of M1/M2 macrophage polarization in DN progression has been paid more attention (27, 28). M1 macrophages produce large amounts of pro-inflammatory cytokines iNOS, TNF-α, MCP-1, and other pro-inflammatory mediators that amplify inflammation, resulting in further damages during DN pathogenesis (29, 30). On the other hand, M2 macrophages inhibit renal inflammation and ameliorate injury by secreting anti-inflammatory cytokines, such as IL-10 and Arg-1 (31, 32).

Accumulating evidence has suggested that the levels of proinflammatory factors (proinflammatory cytokines and chemokines) increase with the development of DN and are independently associated with urinary albumin excretion in DN (33, 34). Proinflammatory cytokines that are synthesized and secreted by macrophages in the local microenvironment can directly damage the renal architecture and then trigger the renal fibrosis (35). Therefore, regulation of M1/M2 macrophage phenotypes exhibits anti-proteinuric and renoprotective effects on DN progression (36–38). However, there have been few studies exploring drugs that can regulate macrophage polarization in DN. In this study, we demonstrated that HPS effectively regulated macrophage polarization by shifting proinflammatory M1 macrophages to M2 ones, resulting in the inhibition of proinflammatory macrophage infiltration in the kidney, and thus altered the balance of pro-inflammatory and anti-inflammatory cytokines in *db/db* mice and in BMDMs *in vitro*. Taken together, our results indicated that the anti-inflammatory effects of HPS *via* the regulation of M1/M2 macrophage polarization may be critical for the direct attenuation of proteinuria and improvement of renal tissue damage.

As reported previously, the inflammation and its progression result from not only innate immune responses dominated by macrophage-mediated effects, but also the adaptive immune responsiveness mediated by lymphocytes. Consistently, in addition to the regulation of macrophage polarization, our study showed that HPS also altered the balance of Th1/Th17/Treg cells. Th1 and Th17 cells have been positively associated with renal damages in DN (39). The hallmark of Th1 and Th17 cells is the production of two cytokines interferon γ (IFNγ) and interleukin (IL)-17, which are abundant in diabetic kidneys and play important roles in the development and progression of inflammatory injury in DN (40, 41). Targeting Th17 cells by mycophenolate mofetil or IL-17A neutralizing antibody could attenuate albuminuria and tubulointerstitial fibrosis in mice with DN (6, 42). On the other hand, Treg cells expressing a specific transcription factor forkhead box P3 (FoxP3) have been implicated in the inhibition of DN progression by suppressing the activation of effector T-cells and exerting anti-inflammatory activity (43). Depletion of Tregs exacerbated diabetic-associated renal injury in *db/db* mice, whereas the adoptive transfer of Tregs or induction of Tregs had the opposite effect (44–48). Studies have reported that Th17/Treg imbalance contributed to the development and progression of DN (41, 45, 49), and reversing the imbalance by Dapagliflozin attenuated albuminuria and tubulointerstitial fibrosis independently of glycemic control in *db/db* mice (50). Therefore, the renoprotective effects of HPS in this study may be also associated with its reversal of Th1/Th17/Treg imbalance.

Interaction of renal tissue macrophages with T cells produces various reactive oxygen species, proinflammatory cytokines, metalloproteinases and growth factors, which in turn enhance the local immune responses and increase inflammation within the kidney in DN (7, 51, 52). Given that HPS remarkedly promoted M2 macrophage polarization in HG-induced BMDMs, we performed co-culture of M2 macrophages and T cells and found that HPS could enhance the ability of M2 macrophages to promote T cell differentiation into Treg and Th2 subsets. Regulation of T-cell proliferation and differentiation by macrophages are well documented in various disease settings, including DN (29, 53, 54). Although the mechanisms of crosstalk between these cells are not clear, it has been reported that M2-polarized macrophages can produce Th2-type and anti-inflammatory cytokines that in turn inhibit T-cell proliferation (55). Therefore, the renoprotective effects of HPS may be attributed to its contribution to the shift of macrophage polarization towards an anti-inflammatory M2 phenotype, which then modulate Th1/Th2 or Th17/Treg balance and thus suppress renal inflammation.

In conclusion, we demonstrated that HPS ameliorates renal inflammatory injury in DN *via* promoting macrophage polarization from an M1 to M2 phenotype and CD4^+^ T cell differentiation into Th2 and Treg populations. Moreover, HPS may enhance the ability of M2 macrophages to suppress inflammation by indirectly promoting T cell differentiation into Th2/Treg subsets. Our findings may be implicated for the treatment of DN in clinic. Further studies on how HPS modulates macrophage polarization are warranted.

## Data Availability Statement

The raw data supporting the conclusions of this article will be made available by the authors, without undue reservation.

## Ethics Statement

The animal study was reviewed and approved by the Institutional Animal Care and Use Committee of Guangzhou University of Chinese Medicine.

## Author Contributions

LZ and XL acquired funding for this research. LZ contributed to the conception and design of the experiments, and analysis and interpretation of the data. LZ and JL drafted this manuscript. JL, YZ, and HS contributed to the performance of the experiments and the acquisition and analysis of data. HL and CL contributed to providing specific input relating to the cell experiment. JG contributed to analyzing the renal morphologic results. ZL contributed to the performance of the animal experiments. WM contributed to providing experimental suggestions. ZD and XL contributed to the design of the experiment and edited this manuscript. All authors contributed to the article and approved the submitted version.

## Funding

This project was supported in part by grants from the State Key Laboratory of Dampness Syndrome of Chinese Medicine (SZ2021ZZ16, SZ2021ZZ43, and SZ2021ZZ18), Guangdong Provincial Key Laboratory of Clinical Research on Traditional Chinese Medicine Syndrome (ZH2020KF02), National Natural Science Foundation of China (81603717, 81873261), Guangzhou University of Chinese Medicine (Grant No: AFD018171Z11099), and Special Fund of Guangdong Provincial Science and Technology Innovation Strategy(Guangdong-Hong Kong-Macau Joint Lab, 2020B1212030006).

## Conflict of Interest

The authors declare that the research was conducted in the absence of any commercial or financial relationships that could be construed as a potential conflict of interest.

## Publisher’s Note

All claims expressed in this article are solely those of the authors and do not necessarily represent those of their affiliated organizations, or those of the publisher, the editors and the reviewers. Any product that may be evaluated in this article, or claim that may be made by its manufacturer, is not guaranteed or endorsed by the publisher.
