# Supplementary material for: Hyperoside Suppresses Renal Inflammation by Regulating Macrophage Polarization in Mice With Type 2 Diabetes Mellitus
Source: Front Immunol. 2021 Dec 3;12:733808. doi: 10.3389/fimmu.2021.733808 (PMC8678409; doi:10.3389/fimmu.2021.733808)
Supplement: Supplementary file 1 [file DataSheet_1.pdf]

Supplementary Materials

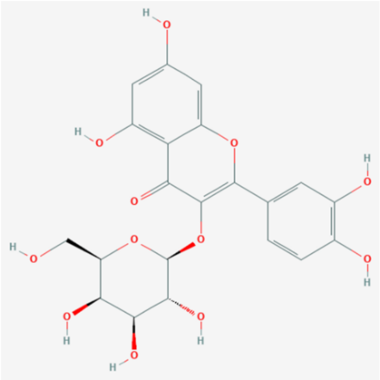

Supplementary Figure 1. Chemical structure of hyperoside

(https://pubchem.ncbi.nlm.nih.gov/compound/5281643)

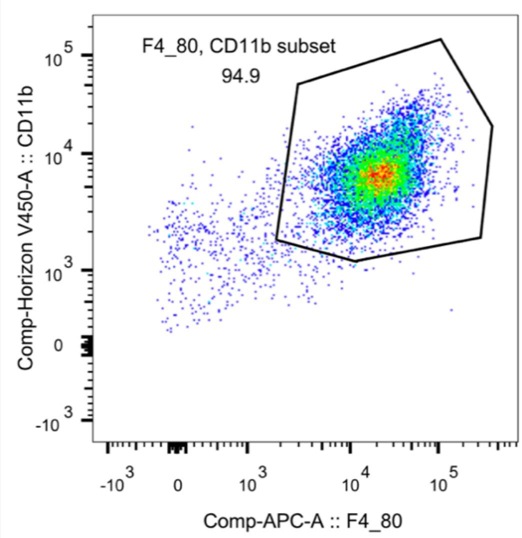

Supplementary Figure 2. The purity of BMDMs as determined via flow cytometry
